# High (130 Hz)- and mid (60 Hz)-frequency deep brain stimulation in the subthalamic nucleus differentially modulate response inhibition: A preliminary combined EEG and eye tracking study

**DOI:** 10.1016/j.nicl.2023.103314

**Published:** 2023-01-05

**Authors:** Josefine Waldthaler, Alexander Sperlich, Aylin König, Charlotte Stüssel, Frank Bremmer, Lars Timmermann, David Pedrosa

**Affiliations:** a Department of Neurology, University Hospital Giessen and Marburg, Marburg, Germany; b Department of Neurology, Philipps-University Marburg, Marburg, Germany; c Center for Mind, Brain, and Behavior (CMBB), Philipps-University Marburg and Justus-Liebig-University, Giessen, Germany; d Department of Neurophysics, Philipps-University Marburg, Marburg, Germany

**Keywords:** Antisaccade, Response inhibition, Parkinson’s disease, Executive functions, Eye tracking, Movement disorders, STN, subthalamic nucleus, DBS, deep brain stimulation, PD, Parkinson's disease, IFG, inferior frontal gyrus, ACC, anterior cingulate cortex, DLPFC, dorsolateral prefrontal cortex, FEF, frontal eye field, SEF, supplementary eye field, TEED, total electrical energy delivered

## Abstract

While deep brain stimulation (DBS) in the subthalamic nucleus (STN) improves motor functions in Parkinson’s disease (PD), it may also increase impulsivity by interfering with the inhibition of reflexive responses. The aim of this study was to investigate if varying the pulse frequency of STN-DBS has a modulating effect on response inhibition and its neural correlates.

For this purpose, 14 persons with PD repeated an antisaccade task in three stimulation settings (DBS off, high-frequency DBS (130 Hz), mid-frequency DBS (60 Hz)) in a randomized order, while eye movements and brain activity via high-density EEG were recorded.

On a behavioral level, 130 Hz DBS stimulation had no effect on response inhibition measured as antisaccade error rate, while 60 Hz DBS induced a slight but significant reduction of directional errors compared with the DBS-off state and 130 Hz DBS. Further, stimulation with both frequencies decreased the onset latency of correct antisaccades, while increasing the latency of directional errors.

Time-frequency domain analysis of the EEG data revealed that 60 Hz DBS was associated with an increase in preparatory theta power over a midfrontal region of interest compared with the off-DBS state which is generally regarded as a marker of increased cognitive control. While no significant differences in brain activity over mid- and lateral prefrontal regions of interest emerged between the 60 Hz and 130 Hz conditions, both stimulation frequencies were associated with a stronger midfrontal beta desynchronization during the mental preparation for correct antisaccades compared with DBS off-state which is discussed in the context of potentially enhanced proactive recruitment of the oculomotor network.

Our preliminary findings suggest that mid-frequency STN-DBS may provide beneficial effects on response inhibition, while both 130 Hz- and 60 Hz STN-DBS may promote voluntary actions at the expense of slower reflexive responses.

## Highlights

• Study on the effect of varying DBS pulse frequency on response inhibition.
• 60 Hz- & 130 Hz-DBS facilitate voluntary actions while impeding reflexive responses.
• 60 Hz- & 130 Hz-DBS are associated with decreased preparatory prefrontal beta power.
• Only 60 Hz-DBS reduces the probability for impulsive actions.
• Higher midfrontal theta power with 60 Hz-DBS indicating enhanced cognitive control.

## 1 Introduction

### 1.1 Response inhibition in Parkinson’s disease

As a core component of executive functions, response inhibition is the ability to withhold prepotent reflexive responses allocating more time to shape the behavioral strategy according to context which often results in more favorable outcomes of our actions (Obeso et al., 2011). Impaired response inhibition, on the other side, leads to impulsivity, i.e., the tendency of acting without delay, reflection or voluntary directing (Bari and Robbins, 2013). Response inhibition is modulated by activity in dopamine-dependent fronto-striatal networks, and is, thus, particularly vulnerable to an aberrant dopaminergic system in Parkinson’s disease (PD) (Kudlicka et al., 2011). In fact, impaired executive functioning as well as impulsive and compulsive behaviors are commonly encountered in PD (Weintraub et al., 2010, Godefroy et al., 2010).

Deep Brain Stimulation (DBS) of the subthalamic nucleus (STN) is an effective treatment in PD. While STN-DBS improves motor symptoms, a variety of behavioral studies have lend credence to stimulation-induced motor impulsivity (Hershey et al., 2004, Ray et al., 2009, Obeso et al., 2013, Witt et al., 2004, Ballanger et al., 2009). In this study, we aim at exploring the effects of different DBS pulse frequencies with respect to switched-off stimulation on the antisaccade task, an established paradigm assessing response inhibition.

### 1.2 Neural correlates of response inhibition

Response inhibition activates a network consisting of prefrontal and premotor regions along with the basal ganglia (Stevens et al., 2007), all of which interact via frequency-specific synchronized neuronal oscillations. In brief, main cortical areas involved in the response inhibition network are the inferior frontal gyrus (iFG), the medial and anterior cingulate cortex (ACC), and the dorsolateral prefrontal cortex (DLPFC). Tasks requiring cognitive control have been consistently associated with theta oscillations (4–8 Hz) over medial frontal regions (referred hereafter as midfrontal theta) (cf. (Cavanagh and Frank, 2014) for review). Midfrontal theta is most likely generated by ACC and the pre-supplemental motor area (pre-SMA) as supported by intracranial recordings in non-human primates and humans as well as fMRI studies (Cohen et al., 2008, Wang et al., 2005, Tsujimoto et al., 2006, Hauser et al., 2014). Since midfrontal theta seems to be causally involved in modulating adaptive cognitive control processes (Van Driel et al., 2015), it has been proposed as neural signature of an action monitoring system of the brain. In PD, midfrontal theta activity is diminished during cognitive control (Singh et al., 2018).

Yet, it is undisputable that assigning cognitive control and response inhibition merely to cortical areas would pose an oversimplification. On a subcortical level, the basal ganglia are critically involved in the process of response inhibition. In particular, and in accordance with classical models of cortico-basal ganglia circuitry, activity in the indirect pathway via STN inhibits prepotent responses to external cues until the selected response is triggered via the direct pathway (Chevalier and Deniau, 1990, Redgrave et al., 1999). These dynamic properties of the STN to delay action selection are pivotal for efficient and successful response inhibition (Herz et al., 2018, Frank, 2006). Frequency-specific STN activity seems to play a role in both reactive inhibition as well as in the implementation of the proactive “hold your horses” signal (Benis et al., 2014). Studies with parallel local field potential (LFP) and EEG recordings suggested that these processes are associated with changes of beta band activity (13–30 Hz) in and synchronization between the iFG and the STN (Schaum et al., 2021, Swann et al., 2011, Alegre et al., 2013), respectively theta band activity in and synchrony between the medial frontal cortex and STN (Zavala et al., 2013, Zavala et al., 2016, Ray et al., 2012) (cf. (Zavala et al., 2015) for review). Growing evidence indicates that chronic STN-DBS may interfere with the inhibition of impulsive motor responses in PD (Frank, 2006, Antonelli et al., 2011, Fluchère et al., 2018). However, the mechanisms underlying this disinhibition remain speculative.

### 1.3 The antisaccade task

**Fig. 1:**
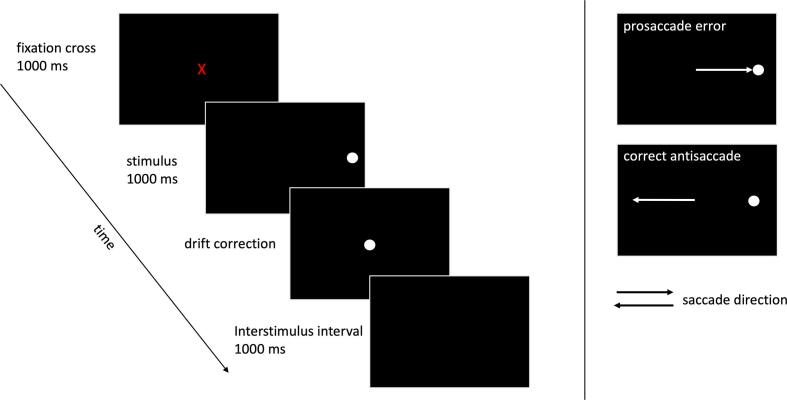
The antisaccade task. Please see section Eye-tracking procedure of Methods for detailed task description.

The antisaccade task is an established eye-tracking paradigm to investigate the ability to inhibit a pre-potent oculomotor response and subsequently exert an alternative action. More precisely, participants are asked to inhibit a reflexive saccade in the direction of a visual stimulus (the *pro*saccade) and to execute a voluntary saccade in the opposite direction instead (the *anti*saccade) (Fig. 1).

Compared with visually-guided prosaccades, the cognitively more demanding antisaccades recruit the right DLPFC and ACC (Brown et al., 2007), whereby higher activation in these areas has been associated with the successful suppression of reflexive prosaccade errors (Pa et al., 2014). Moreover, pre-stimulus mental preparation for antisaccades induces an increase in midfrontal theta power and fronto-central inter-trial theta coherence compared with both no-go trials and errors (van Noordt et al., 2017, Cordones et al., 2013) further supporting the idea of increased top-down cognitive control during the preparation for antisaccades.

Given that desynchronization of beta band oscillations (13–30 Hz) is generally coined as a facilitator of movement initiation, its role during the initiation of saccades is also conceivable (Zhang et al., 2008). In an MEG study, *increased* beta band power over the lateral prefrontal cortex coupled with increased alpha band power over the frontal eye field (FEF) during the preparation for antisaccades compared with prosaccades. Furthermore, higher pre-stimulus alpha power in FEF, which has been interpreted as a correlate of local inhibition, was associated with successful inhibition of a prepotent reflexive error (Hwang et al., 2014).

### 1.4 Antisaccades in PD and the potential influence of DBS frequency

When ask to perform antisaccades persons with PD tend to higher rates of erroneous prepotent saccades towards, instead of away from the stimulus, than healthy controls (van Stockum et al., 2008, Waldthaler et al., 2019, Antoniades et al., 2015). The influence of STN-DBS on response inhibition in the antisaccade task remains under debate. A recent meta-analysis of studies on the effects of STN-DBS on antisaccades in PD concluded that DBS reduces their latency, while a moderate increasing effect on the antisaccade error rate did not reach significance, but was possibly underpowered with only five eligible studies (Waldthaler et al., 2021).

With 130 Hz as the default stimulation frequency, most patients are treated with DBS pulses between 60 and 200 Hz. A differential effect on several clinical hallmarks of PD has empirically evolved with higher frequencies enabling tremor control and lower frequency stimulation (60 to 90 Hz) possibly improving gait function and axial symptoms (Su et al., 2018). The limited evidence to date allows an application of mid-frequency stimulation in individual cases (Conway et al., 2019). Nevertheless, further insight is warranted as higher DBS frequencies (>100 Hz) may have detrimental effects on cognition (Combs et al., 2015), while verbal fluency (Wojtecki et al., 2006) and cognitive interference (Varriale et al., 2018) may, on the contrary, even improve with lower frequency pulses.

Interestingly, axial motor signs, and specifically freezing of gait, paralleled antisaccade performance in several recent trials (Waldthaler et al., 2019, Nemanich and Earhart, 2016, Walton et al., 2015, Ewenczyk et al., 2017, Gallea et al., 2021). Hallmark regions for gait impairment such as the pedunculopontine nucleus correlate in their functional connectivity with FEF, which in turn, correlates with antisaccade latency in an fMRI study (Ewenczyk et al., 2017, Gallea et al., 2021). Thus, a common underlying mechanism of freezing of gait and antisaccade control may be posit as an expression of a network-dependent degeneration (Ruppert et al., 2021). Given a possible modulation of gait with mid-frequency stimulation (Moreau et al., 2008), the question seems pertinent whether 60 Hz-DBS may have an effect on antisaccades as well.

In this study, we combined eye-tracking and EEG recordings with the aim to explore effects of high (130 Hz)- versus mid-frequency (60 Hz) STN-DBS versus no stimulation on response inhibition and its cortical correlates in the antisaccade task in PD.

## 2 Material and methods

### 2.1 Ethical compliance statement

The study was approved by the Ethical Board of the University Hospital Marburg (reference number 119/19) and followed the Declaration of Helsinki. All participants gave written informed consent before participating. Patients were recruited from the Movement Disorders Outpatient Clinic of the Department of Neurology at the University Hospital Marburg.

### 2.2 Participants

A total of 19 consecutive participants suffering from PD according to the clinical diagnostic criteria of the Movement Disorders Society (Postuma et al., 2015) and treated with chronic STN-DBS were recruited. All patients were implanted with bilateral DBS leads targeting the sensorimotor part of STN (Vercise Cartesia™ Directional Lead, Boston Scientific Neuromodulation Corporation, Valencia, CA91355, USA). A minimum of three months between study inclusion and DBS surgery was required to avoid any impact of lesion effects on the results. All patients had undergone an extensive monopolar review to find the optimal settings for DBS minimizing motor symptom and avoiding side effects. Pre-established exclusion criteria were 1) dementia according to the MDS task force criteria level 1 (Emre et al., 2007), 2) signs of clinically relevant depression (Beck Depression Inventory > 14 points), 3) history of other disorders of the CNS, 4) any concurrent conditions making eye-tracking or EEG recordings impossible (e.g., disorders of the eyes or visual system with reduced visual acuity, severe camptocormia, other orthopedic disorders impairing ability to sit for longer periods, etc.), and 5) medications possibly influencing eye movements or EEG recordings. None of the participants received non-dopaminergic medications with cognitively enhancing effects like cholinesterase inhibitors.

All participants were in off-medication state after overnight withdrawal of dopaminergic medication and at least 12 h prior to the start of the assessments. Motor symptoms were rated on part III of the Movement Disorder Society – Unified Parkinson’s Disease Rating Scale (MDS-UPDRS) (Goetz et al., 2007). Levodopa equivalent daily doses were calculated according to (Tomlinson et al., 2010). Montreal Cognitive Assessment (MoCA) was used to evaluate general cognitive ability (Nasreddine et al., 2005).

The final data set included 14 participants. Five of a total of 19 recruited participants had to be excluded from the analysis since they had asked for pre-mature stopping of the study protocol due to tiredness, unbearable motor symptoms or pain before completion of at least one antisaccade block in all three conditions. Please see Table 1 for a summary of demographic and clinical characteristics.

**Table 1:** Demographics and clinical characteristics of the PD group.

**age (years), mean (sd)**	57.0 (8.8)
**sex, n (%) female**	4 (29 %)
**symptom lateralization, n (%) right**	5 (36 %)
**disease duration (years), mean (sd)**	8.6 (3.3)
**time since DBS surgery (months), mean (sd)**	10.2 (9.1)
**LEDD (mg), mean (sd)**	476 (307)
**MDS-UPDRS III OFF/OFF, mean (sd)**	38.9 (9.9)
**MDS-UPDRS III OFF/ON, mean (sd)**	15.7 (8.7)
**MoCA, mean (sd)**	26.5 (2.3)

### 2.3 DBS programming

After overnight-withdrawal from dopaminergic medication, participants performed the antisaccade task (Fig. 1) three times: i.) with DBS switched off, ii.) with DBS frequency set at 130 Hz and iii.) with DBS frequency set at 60 Hz. All participants completed recording sessions in all three DBS conditions on the same day. The order of conditions was randomized, and participants were blinded to avoid any biases due to expectation, learning effects or tiredness.

All other DBS parameters (contacts, amplitude, and impulse width) remained unaltered with respect to the chronic DBS program with optimal clinical response in each individual patient (Supplementary Material 1). The participants were blinded for the active DBS program and there were wash-out periods of at least ten minutes between sessions. MDS-UPDRS III was assessed directly prior to the EEG recordings in each DBS condition.

There are recommendations to keep the total electrical energy delivered (TEED) constant between DBS programs by adjustments of stimulation amplitude when changing the pulse frequency (Moro et al., 2002). On the other hand, some authors discourage the use of TEED to censor or edit combinations of stimulation parameters (Marks, 2015). We decided against adjustments since the physiological role of TEED is subject of debate and increasing amplitudes in the 60 Hz condition (given that most participants were treated at higher frequencies) might have introduced additional bias or may have caused side effects (Koss et al., 2005).

Recently, effects of even lower stimulation frequencies in the theta or delta range on cognitive performance have been reported (Wojtecki et al., 2006, Lee et al., 2021). To avoid confusion with these low DBS frequencies, we decided to refer to the 60-Hz condition as mid-frequency stimulation throughout this manuscript.

### 2.4 Eye-tracking procedure

The experiment took place in a sound-attenuated, darkened and electrically shielded room which the researcher monitoring the progress in the adjoining room. All participants were seated in an upright armchair with back support at distance of 70 cm from a computer monitor with a diagonal of 60 cm and with their head stabilized with chin and forehead rests.

An infrared video-based eye-tracker (EyeLink 1000 Plus, SR Research, Ontario, Canada) recorded positions of both eyes at a sampling rate of 500 Hz and an instrumental spatial resolution of 0.01° with simultaneous recording of EEG data. The eye-tracker was calibrated and validated with a 9-point grid before each experimental block. The validation was repeated until average errors for all points were < 1° compared to the result of the calibration. Moreover, to ensure precision within blocks, a drift correction prior to each trial was performed.

The experiment was programmed in MATLAB 2020b (The Mathworks Inc., Massachusetts, USA) using the psychophysics toolbox (https://www.psychtoolbox.org) (Brainard, 1997). Three blocks of 50 horizontal antisaccades each were presented per condition (n = 150 per condition).

Each trial started with a red central fixation cue (diameter 1° visual angle) that was presented for 1000 ms in the middle of a black screen. It was followed by the appearance of a white lateral target stimulus located either 10° left or right from the initial fixation cue (Fig. 1A). The lateral stimulus was presented in equal numbers and random order to the left and right side of the screen. It vanished after 1000 ms and was followed by a white central dot for drift correction and a subsequent interstimulus interval (blank black screen) that allowed participants to blink. The next trial started with a new red central fixation cue.

The participants were instructed to look at the exact opposite direction of the lateral stimulus as fast *and* precisely as possible as soon as it was presented on the screen. Ten practice trials prior to the first antisaccade block of the experiment with verbal feedback ensured that participants understood the instructions. These practice trials were discarded. Between blocks, participants were given the opportunity to take breaks.

### 2.5 Eye-tracking data processing and analysis

The researcher analyzing the eye-tracking and EEG data sets (JW) was not involved in data collection and was blinded to the participants’ identities. A parsing system incorporated in the EyeLink 1000 software intersected the raw eye position data into visual events, i.e., saccades, fixations, and blinks. This event data set was analyzed in the statistical computing program R (R Core Team, 2014) using the Eyelinker package. An acceleration >8000°/s2 and a velocity > 30°/s were set as thresholds for saccade detection.

Saccade latency was defined as the time from stimulus onset to the start of the first saccade regardless of whether the saccade was elicited in the correct direction. A directive error was defined as a saccade towards lateral stimuli, i.e., a prosaccade. Trials were removed from further analysis when i) the latency was in the anticipatory range (<90 ms) or longer than two standard deviations from the individual mean latency of the participant, ii) the first saccade after stimulus onset had a starting position >3° lateral of the central fixation cue, iii) a saccade with an amplitude smaller than 0.5° or >15° was executed or iv) a blink occurred between stimulus presentation and the first saccade. Processing of the eye-tracking data led to the rejection of a total of 16.8 % ± 11.5 % of all recorded trials (off: 19.7 % ± 14.3 %; 130 Hz: 14.4 % ± 11.3 %; 60 Hz: 16.2 % ± 8.6 %, χ2(2, 13) = 3.964, p = 0.1).

### 2.6 EEG recording

EEG was recorded simultaneously during the eye-tracking sessions described above. We used an elastic cap with 128 electrodes mounted in a spherical array (Easy-Cap GmbH, Herrsching, Germany). To maintain electrode impedances below 10 kΩ, conduction gel was applied. The used caps were standardized and placed according to the 10/10 system. All data were recorded on a BrainAmp® standard amplifier (Brain Products GmbH, Gilching, Germany), low-pass filtered at 1 kHz and digitized at a sampling rate of 5 kHz. In addition to the scalp EEG electrodes, an electrocardiogram electrode was placed for recording of cardiac activity.

### 2.7 EEG preprocessing

Four participants had to be excluded from the EEG analyses due to technical failure during the recordings resulting in a total of ten participant whereby the behavioral results in this subgroup did not differ from the entire sample of 14 participants.

EEG data processing and statistical analysis were run in MATLAB 2020b (The MathWorks Inc., Massachusetts, USA) and MNE Python (Gramfort et al., 2013) with Python version 3.7. First, data was resampled at 250 Hz, re-referenced to average and high-pass filtered to remove DC offset and drift (4th-order Butterworth filter, cut-off frequency 0.5 Hz). DBS artefacts were removed using the DBSFilt toolbox (https://github.com/guillaumelio/DBSFILT/blob/master/DBSFILT_GUI_DOC.pdf) which, briefly, filters the EEG signal and detects spikes based on the Hampel identifier for automated spike detection (Allen, 2009). This identifier treats artefacts as outliers in the frequency domain and replaces them with interpolated values, which was successfully used for DBS artefact removal before (Allen et al., 2010).

Additional EEG artefacts were detected and discarded as follows: First, bad channels were identified visually and corrected with the spherical spline method, which projects the sensor locations onto a unit sphere and interpolates the signal at the bad sensor locations based on the signals at surrounding artefact-free locations (Perrin et al., 1989). Consecutively, an independent component analysis (ICA) was used for blink as well as eye movement and heart artefact correction (3.2 ± 0.8 components removed) (Delorme et al., 2007).

Data were cut into epochs ranging from 500 ms before fixation cue onset to 100 ms after stimulus onset to allow edge artifacts to subside outside the actual window of interest. Only epochs in which a subsequent correct antisaccade was performed were further analyzed. Trial rejections during eye-tracking and EEG preprocessing resulted in an average number of correctly executed antisaccade trials of 68 ± 49 in the DBS-off state, 79 ± 40 in the 130 Hz-DBS and 80 ± 32 for 60 Hz-DBS condition remaining for time–frequency analysis. For statistical testing, the number of trials was randomly equalized between all three conditions within each subject using the “equalize_epoch_counts” function implemented in MNE Python to maintain a constant signal-to-noise-ratio within subjects.

### 2.8 Time-frequency analysis

Based on our *a priori* hypothesis, we restricted sensor-level EEG analyses to a selection of frontal EEG electrodes to avoid unnecessary multiple comparison. To focus on the hypothesized role of midfrontal theta oscillations during periods of enhanced cognitive control and during the preparatory period for an antisaccade in particular (Zavala et al., 2016, van Noordt et al., 2017, Cordones et al., 2013), time–frequency data from a midfrontal region of interest (ROI) encompassing the electrodes *F1, Fz* and *F2* were averaged. As right-lateralization of dynamics in DLPFC and iFG has been a recurrent finding in studies on response inhibition and antisaccade preparation (Swann et al., 2011, Hwang et al., 2014, Hamm et al., 2012), we also defined a right lateral prefrontal ROI including the electrodes AF8, F6, F8, and FC6. All further analyses were restricted to these two ROIs.

For each condition, time–frequency representations (TFR) of oscillatory power changes resulted from a Morlet wavelet decomposition with variable, frequency-dependent cycles (=frequency / 2) into frequency bins between 3 and 30 Hz. The length of the wavelets increased linearly from 1 cycle at 2 Hz to 15 cycles at 30 Hz to optimize the trade-off between temporal resolution at lower frequencies and stability at higher frequencies. The time window from 200 ms to 100 ms before presentation of the fixation cue was defined as baseline (-1200 ms to −1100 ms). The baseline was offset by 100 ms from fixation cue onset to minimize contamination of the baseline interval by fixation-associated activity. The change in spectral power during the preparatory period (−1000 ms to 0 ms) is reported as the logratio from the baseline period, calculated by dividing by the mean baseline power per frequency and converting to decibel (dB) by log-transformation (dB = 10 × log10(power/baseline), then averaged across trials for each condition.

Since this study focused on preparatory activity and eye movements were inherent in the response period of the trials, the final epochs were limited to the time window before stimulus presentation, i.e., before the direction of the following saccade had been revealed to the participant. Thus, data were segmented into epochs time-locked to the onset of the cue stimulus containing the full 1000 ms period of fixation cue presentation (−1000 ms to 0 ms with respect to stimulus onset) for statistical analysis. (Fig. 1). In this way, eye movement artefacts as well as any brain activity related to the sensorimotor transformation of the stimulus into a saccade (Moon et al., 2007) were excluded.

To investigate effects of DBS conditions on cortical activity on a single-trial level, time–frequency transformations were adapted for single-trial analysis by calculating TFR for each trial separately using the same Morlet wavelet decomposition and baseline correction as outlined above. For the construction of the generalized mixed-effect models that were used to predict trial outcome (correct antisaccade / error), TFR were additionally calculated for error trials, while only correct antisaccade trials were included in the single-trial mixed linear models for antisaccade latency. For the single-trial analysis, frequency ranges and time windows were determined post-hoc based on the group level results (see Results section).

### 2.9 Statistical analysis

Generalized mixed models were constructed to compare error rates and latency of correct antisaccades and errors between the three conditions using the R package lme4 (Bates, 2005). Condition-averaged approaches would have under-represented the high variability in both trial numbers and behavioral outcomes. In contrast, generalized mixed-effect modeling is a robust statistical method for the repeated measure design of this study accounting for the inherent correlation between repeated measures from each participant (Wainwright et al., 2007). A mixed-effect linear model was run to assess the relationship between conditions and latencies on a single trial level with condition and trial outcome (correct antisaccade / error) as well as their interaction as fixed effects and participant as random effect. Likewise, to assess the effect of condition of the antisaccade outcome (correct antisaccade / error), a mixed model logistic regression was constructed with main effect of condition as fixed effect and participants as random effect. The resulting coefficients were deemed significant by the Satterthwaite approximation (Kuznetsova et al., 2016). Effect sizes were calculated as Cohen’s d as described in (Westfall et al., 2014). P-values of the pairwise comparisons between the three conditions were Bonferroni-corrected to account for multiple comparison.

Statistical inference of the EEG data was ascertained with cluster-based permutation tests implemented in MNE Python (Maris and Oostenveld, 2007). This approach corrects for multiple comparisons within time–frequency representations by identifying clusters of differences between conditions by summing adjacent significantly different time–frequency bins and comparing the cluster size to a distribution of largest cluster values obtained by randomly shuffling the conditional labels under the null-hypothesis. If the observed cluster statistics exceeded 95 % of the permutation distribution (corresponding to critical α = 0.05) the null hypothesis was rejected. Cluster-based permutation one-way repeated-measures analysis of variance (RM-ANOVA) with 1000 permutations was used to compare the time–frequency representations between the three DBS conditions (off / 130 Hz / 60 Hz) followed by pairwise comparisons between the conditions using cluster-based permutation paired-t-tests with 511 permutations (exact full permutation test).

To assess whether preparatory beta or theta activity may predict the antisaccade outcome (correct antisaccade / error) or antisaccade latency on a single trial level, a second set of generalized mixed-effect models were run with main effects of condition and theta, respectively beta activity (cf. Results section) as well as their interaction as fixed effects and participants as random effect.

### 2.10 Data availability

The raw eye-tracking and EEG datasets are available upon reasonable request from the corresponding author. All newly generated custom-written code for the task (Matlab) and analysis pipelines (R, Matlab, Python) is available on the following GitHub page: https://github.com/JoWld/PD_DBS_Antisac.

## 3 Results

### 3.1 Demographics

Fourteen participants with PD and STN-DBS with an average time between study inclusion and implantation of DBS leads of 10.2 ± 9.1 months were included in the final analysis. See Table 1 for demographics and clinical characteristics.

### 3.2 Behavioral results of the antisaccade task

A logistic mixed-effect model evaluating the relationship between DBS condition and trial outcome with participants as random effect revealed a significant effect of condition on trial outcome. 59.1 % of all trials were performed correctly in the 60 Hz-DBS condition, 54.4 % in 130 Hz-DBS and 51.4 % in off-DBS state. The effects of 60 Hz-DBS and off-DBS state (Odds Ratio (OR) = 1.54, 95 %-CI = [1.31, 1.81], p_adj_ < 0.001), respectively 60 Hz-DBS and 130 Hz-DBS (OR = 1.44, 95 %-CI = [1.23, 1.69], p_adj_ < 0.001) on trial outcome were significantly different, while there was no difference between 130-Hz DBS and off-DBS state (OR = 1.07, 95 %-CI = [0.91, 1.25], p_adj_ = 0.8). Thus, 60 Hz-DBS increased the probability for the execution of a correct antisaccade compared with 130 Hz-DBS and off-DBS state.

In the generalized linear mixed-effect model evaluating the relationship between DBS condition, trial outcome and antisaccade latency with participants as random effect, we observed a strong main effect of trial outcome (χ^2^(2) = 1279.2, p < 0.001) on the saccade latency with errors executed with a mean latency of 228 ms compared with 352 ms in correct antisaccades. There was no main effect of condition (χ^2^(2) = 3.121, p = 0.2) on antisaccade latency. However, the interaction effect between condition and trial outcome was significant (χ^2^(2) = 44.685, p < 0.001). The effects of both 130-Hz DBS (t(5194) = -6.658, p_adj_ < 0.001) and 60 Hz-DBS conditions (t(5194) = -4.004, p_adj_ < 0.001) differed from the off-DBS state indicating that the effect of DBS frequency on the latency varies between correct antisaccades and errors. Antisaccade latency was significantly decreased by 21.6 ms (95 %-CI = [-29.3, −13.8], d = 0.256, p_adj_ < 0.001) with 130 Hz-DBS and by 11.9 ms (CI = [-19.7; −4.2], d = 0.142, p_adj_ = 0.1) with 60 Hz-DBS compared with off-DBS state. The latency of errors, on the other hand, increased by 12.7 ms (CI = [6.9; 18.5], d = 0.214, p_adj_ < 0.001) with 130 Hz-DBS and by 8.7 ms (CI = [2.6; 14.7], d = 0.146, p_adj_ = 0.02) with 60 Hz-DBS compared with off-DBS state. There were no significant differences between the two pulse frequencies.

**Fig. 2:**
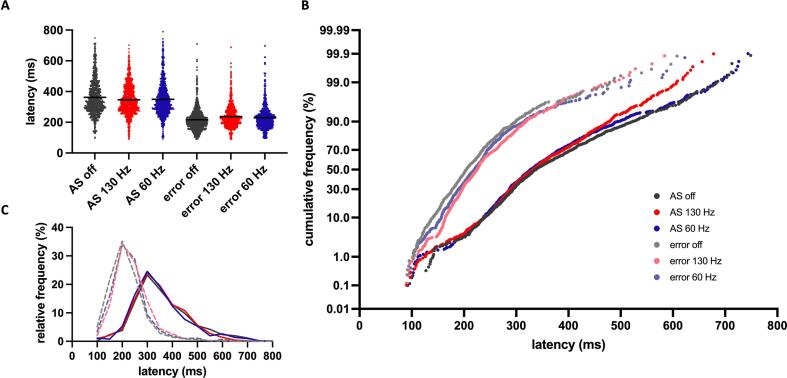
**A**: Dot plots showing latencies of all antisaccade (AS), and error trials pooled for all participants. Black horizontal lines represent the mean. **B**: Cumulative frequency distributions of latencies with probit scaled y axis. Correct antisaccades (AS) displayed in intense colors and errors in faint colors. **C**: Relative frequency distributions of latencies in bins of 50 ms. Solid lines represent correct antisaccade trials, dashed lines represent errors.

To further visualize the distribution of latencies across trials, the relative and cumulative latency distributions of all trials are shown color-coded for the three conditions (Fig. 2). A prominent feature in the off-DBS condition compared with 130 Hz and 60 Hz-DBS was a high proportion of early error saccades within the express (<130 ms) and very fast reflexive (<150 ms) range.

### 3.3 Preparatory midfrontal EEG dynamics

**Fig. 3:**
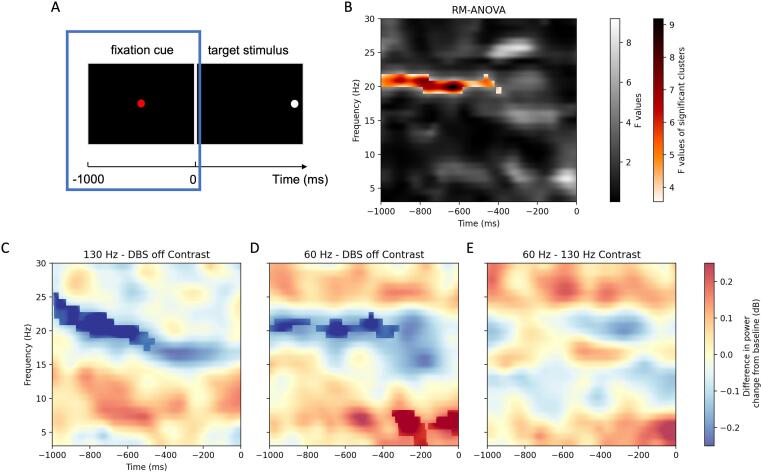
**A**: The time window of the TFR analysis corresponds to the presentation of the fixation cue during the antisaccade trial. **B:** Results of the RM-ANOVA comparing the averaged time–frequency representations in the midfrontal region of interest (Fz, F1, F2) between the three DBS conditions. Highlighted is the cluster of significant differences in power change that led to the rejection of the null hypothesis. Non-significant F values in sequential gray, F values corresponding to the cluster of significant group difference in color. **C-E:** Time-frequency representations of the contrasts between conditions. Bold colors highlight the significant clusters in the pairwise comparisons.

The results of a one-way repeated-measures analysis of variance (RM-ANOVA) with permutation clustering comparing the time–frequency representations (TFR) in the midfrontal region of interest (ROI) between the three DBS conditions are presented in Fig. 3.

RM-ANOVA showed a significant cluster indicating a main effect on beta power (18 – 22 Hz) in the midfrontal ROI during the early preparatory period (p = 0.04) (Fig. 3B). Pairwise comparisons revealed that this effect was driven by a larger decrease in beta power (18–26 Hz) from baseline between −1000 ms and approximately −500 ms in 130 Hz-DBS (p = 0.01) and between −1000 ms and approximately −300 ms in 60 Hz-DBS (p = 0.04) compared with off-DBS state (Fig. 3C-E).

A second significant cluster indicating a theta effect (4 – 8 Hz) during the second half of the preparatory period from approximately −400 ms to stimulus onset at 0 ms was observed in pairwise comparisons between the 60 Hz-DBS condition and DBS-off state (p = 0.04) (Fig. 3D).

For the pre-defined frontolateral ROI encompassing the right lateral prefrontal cortex, RM-ANOVA with permutation clustering resulted in no significant differences of TFR between DBS conditions (Supplementary Material 1).

### 3.4 Condition-dependent single-trial predictive value of midfrontal theta power

Based on the results above, we restricted the single-trial analysis to the frequency ranges (beta: 18–26 Hz, theta: 4–8 Hz) and time windows (beta: −1000 ms to −300 ms, theta: −400 ms to 0 ms) for which significant effects of DBS condition were identified in the group level analysis.

In the linear mixed model evaluating the relationship between midfrontal theta power, DBS condition and antisaccade latency with participants as random effect, we observed a main effect of condition on antisaccade latency as expected from behavioral findings (χ^2^(2) = 33.39, p < 0.001). There was no main effect of theta power on antisaccade latency (χ^2^(1) = 0.942, p = 0.3). However, the interaction effect between condition and theta power was found to be significant (χ^2^(2) = 7.327, p = 0.03), with 130-Hz DBS differing from the off-DBS state (β = 0.112, 95 %-CI = [0.03, 0.19], d = 0.352, t(1654) = 2.703, p_adj_ = 0.01), indicating that the effect of theta activity on antisaccade latency varies between these two conditions.

**Fig. 4:**
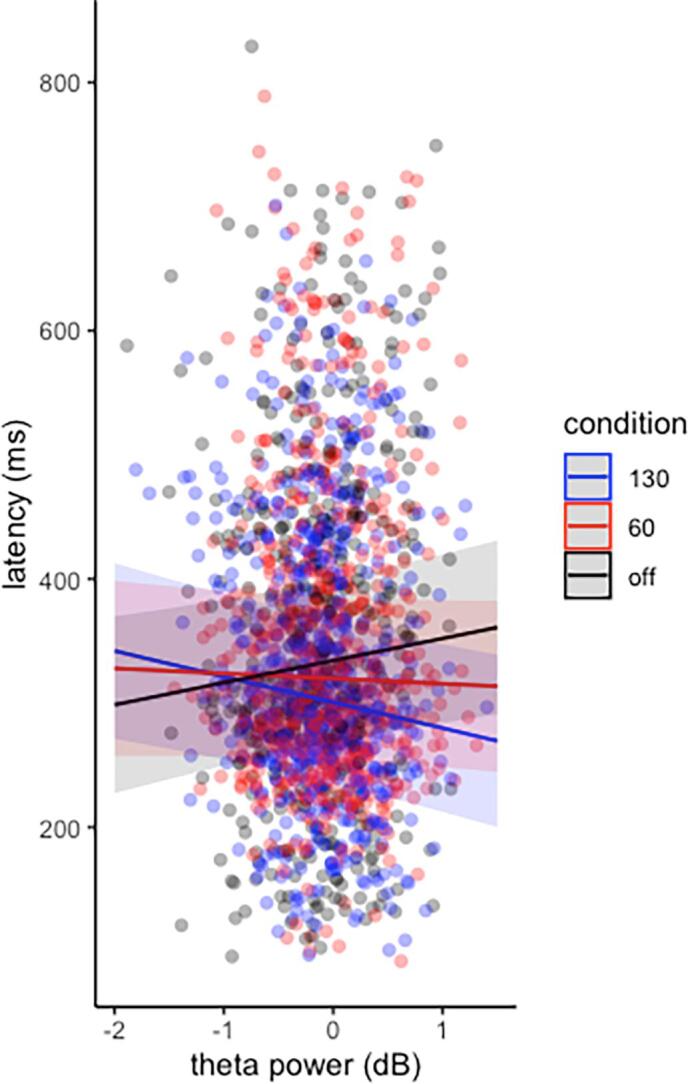
Single-trial mixed linear regression model of the relationship between antisaccade latency (in ms), midfrontal-theta activity (in dB) and DBS condition (off-DBS in black, 130 Hz DBS in red and 60 Hz-DBS in blue) as fixed effects and participants as random effect. Dots represent single trials. Shaded areas represent 95 %-confidence intervals. The (theta × condition) interaction differed significantly between off-DBS and 130 Hz DBS. (For interpretation of the references to color in this figure legend, the reader is referred to the web version of this article.)

From Fig. 4, it is evident that as theta increased, antisaccade latency increased in off-DBS state, while it decreased with 130 Hz-DBS. Thus, 130 Hz-DBS reversed the effect of midfrontal theta activity on antisaccade latency.

No significant effects of cortical power nor of (power × condition) interactions were identified in the linear mixed-effect model including beta power or in the respective mixed logistic models of error probability. For complete results, please see Supplementary Material 1.

## 4 Discussion

### 4.1 Summary of findings

In this study, we aimed at investigating the effects of high- and mid-frequency STN-DBS on response inhibition in patients with PD using the antisaccade task in combination with EEG recordings. Against our a priori hypothesis, our behavioral findings did not support that high-frequency STN-DBS leads to a higher probability of impulsive actions in the antisaccade task in PD. Sixty Hz-DBS may even have a beneficial effect on response inhibition as it was related to a higher accuracy in the antisaccade task compared with both off-DBS state and high-frequency stimulation.

While no significant differences in mid- or lateral prefrontal brain activity emerged between the 60 Hz and 130 Hz conditions in the time–frequency domain analysis of the EEG data, comparison with DBS off-state revealed that 60 Hz-DBS may be associated with an increase in midfrontal theta band power during the pre-stimulus period. In off-DBS state, higher midfrontal theta activity was associated with longer antisaccade latency in a trial-wise analysis. This relationship was reversed by 130 Hz-DBS, indicating that theta-based preparatory activity may be modulated by high frequency STN-DBS.

Switching on STN-DBS had opposing effects on the latency of correctly executed antisaccades and erroneous reflexive saccades regardless of pulse frequency. While antisaccade latency decreased, the latency for directional errors towards the visual stimulus increased in both DBS conditions. During the preparatory period before stimulus presentation, high- and mid-frequency STN-DBS both induced a decrease of midfrontal beta power compared with off-DBS state.

### 4.2 No evidence for impulsivity in the antisaccade task under STN-DBS

Stimulation with 130 Hz- and 60 Hz-pulses led to a reduction of antisaccade latency which is in line with previous studies investigating the effect of DBS on the antisaccade task without considering the pulse frequency (Yugeta et al., 2010, Rivaud-Péchoux et al., 2000, Bakhtiari et al., 2020). Against our hypothesis, 130 Hz-DBS had no detrimental effect on the error rate, while 60 Hz-DBS was, in fact, associated with a decreased error probability. However, we have to acknowledge that the effect size was small and its relevance in clinical practice still needs to be determined. In sum, we found no evidence for an association of high- or mid-frequency STN-DBS with increased motor impulsivity.

The effect of DBS on latencies was opposing with correct antisaccades (reduced) and reflexive prosaccade errors (increased). Thus, STN-DBS may thus result in specific promotion of voluntary actions (a correct antisaccade) accompanied by a deceleration of the reflexive response (a prosaccade error) rather than a general acceleration of saccade initiation. This result opposes previous findings supporting increased motor impulsivity with STN-DBS (Scherrer et al., 2020). However, the response inhibition tasks used in most of these studies bear on the reactive cancellation of all ongoing actions (e.g., the stop signal task) on which STN-DBS seems to exert detrimental influence. In contrast, the antisaccade task relies on *proactive and selective* response inhibition prior to the initiation of the selected response. In line with our findings, the notion that STN-DBS may differentially modulate reactive and proactive, respectively general and selective aspects of response inhibition has previously been discussed (Obeso et al., 2013, De Pretto et al., 2021).

### 4.3 Attenuation of preparatory beta power as a proactive mechanism

Since beta desynchronization is generally coined a facilitator of movement initiation and of changing the ongoing motor set, the attenuated pre-stimulus beta activity under DBS pulses at 130 and 60 Hz alike suggests higher levels of early proactive activation regardless of stimulation frequency. In line with this interpretation, healthy individuals also show prefrontal pre-stimulus beta desynchronization in antisaccades when contrasted with no-go trials (Cordones et al., 2013). As such, our data is consistent with current theories postulating an anticipatory, proactive role of beta power modulation for the preparation for motor and cognitive responses (Jenkinson and Brown, 2011, Oswal et al., 2012).

In PD, a lack of preparatory beta desynchronization has been interpreted as a general shift from proactive to more reactive motor control (Praamstra and Pope, 2007, Te Woerd et al., 2015). Consistent with our findings, STN-DBS may attenuate this aberrant cortical beta activity (Abbasi et al., 2018, Devos et al., 2004) which may, in turn, facilitate proactive control of (eye) movements.

At the same time, stability of beta oscillations also facilitates motor inhibition, so that its attenuation may result in a higher probability of errors. In this regard, Hamm and colleagues showed that preparatory beta power in ACC was lower for errors than for correct antisaccade trials in healthy individuals (Hamm et al., 2012). The authors argued that tonic beta activity may be crucial for correct antisaccade execution as it prevents errors by maintaining the ongoing oculomotor set (i.e., a fixation). Notably, enhanced beta desynchronization with STN-DBS in our study was found in trials with a subsequent successful antisaccade. Thus, a certain level of beta suppression might be necessary to permit the dynamic reconfiguration of neural networks into a state of readiness for executive processing (Oswal et al., 2012). Supporting this interpretation, no significant detrimental behavioral effect of the stimulation on the probability of errors was evident in our cohort. Therefore, we hypothesize that STN-DBS normalized the amount of preparatory beta desynchronization, which had been diminished in the off-medication and off-DBS state (Singh, 2018), allowing sufficient proactive preparation of the oculomotor network without causing impulsive responses. Nevertheless, the relationship between prefrontal beta activity and antisaccade outcome may differ after the stimulus direction has been revealed. In fact, successful response inhibition has been associated with an increase in prefrontal beta power only after the stimulus has been presented, but not during the cue period (Liebrand et al., 2017). For instance, *higher* beta band power over the right prefrontal cortex around the time of stopping an ongoing motor response has been identified under STN-DBS compared with the off-DBS state, and associated with improved response inhibition (Swann et al., 2011). Since we did not analyze the changes in cortical oscillations after stimulus presentation, any further considerations on potential changes of beta power later in the trial are beyond the scope of this study.

### 4.4 Midfrontal theta power and response inhibition

Midfrontal theta activity reflects cortical correlates of cognitive control which may be exerted via synchronized activity in a prefrontal-subthalamic network (Zavala et al., 2016). It has been proposed that medial frontal cortical areas, e.g. the ACC, activate the STN to inhibit impulsive actions via theta oscillations as soon as conflicts arise or the need for cognitive control is detected (Zavala et al., 2014). Further, ACC has been shown to have top-down control over the frontoparietal oculomotor network during the preparatory period for antisaccades, supported by a strong theta and beta synchronization from ACC to FEF (Babapoor-Farrokhran et al., 2017). Together, these preparatory oscillatory changes may subsequently prevent an early reflexive prosaccade (that is an error) when the stimulus is presented, thereby allowing additional time needed to activate the correct oculomotor set for a voluntary saccade later in the trial. In healthy individuals, error trials, as compared to correct antisaccades, were associated with a lack of increase in midfrontal theta during the preparatory period (van Noordt et al., 2017).

Since 60 Hz-DBS increased preparatory midfrontal theta activity in contrast to off-DBS state and reduced the probability of reflexive errors compared with both off-DBS state and the 130-Hz condition, our results provide first preliminary evidence that mid-frequency DBS may improve response inhibition by enhancing proactive cognitive control in PD via midfrontal theta oscillations. Needless to say, a causal link between these findings cannot be implied based on the design and findings of our study. Beyond that, no significant correlation between the trial-by-trial outcomes of the behavioral and EEG analysis was detectable. Our interpretation of these findings, hence, needs to be confirmed in the future by larger studies.

### 4.5 The influence of STN-DBS on midfrontal theta power and antisaccade latency

In the single-trial EEG analysis, higher theta activity during the late preparatory phase precited longer antisaccade latency in the off-DBS state. Consistent with this finding, theta activity has been associated with a slowing of the upcoming response in a variety of cognitively demanding tasks (van Driel et al., 2015, Cohen and Cavanagh, 2011, Cooper et al., 2019). Given this relationship in healthy controls and individuals with PD in off-DBS state, one may also expect an increase in antisaccade latency with the increase in midfrontal theta power with 60 Hz-DBS which was, however, not supported by our data.

Conversely, the trial-by-trial correlation analysis revealed an inversion of the relationship between midfrontal theta power and antisaccade latency with 130 Hz-DBS. This finding is in line with results from Cavanagh and colleagues who described the same stimulation-induced inversion of the relationship between midfrontal theta and response times during high conflict trials in a decision-making task (Cavanagh et al., 2011). This reversal was further associated with increased impulsivity which is not the case in our current study.

In the model of striatal action selection, the STN is pivotal for inhibiting prepotent actions under conflict (Zaghloul et al., 2012), i.e., when more than one potential response set are triggered simultaneously and compete to be selected as a response to the same external stimulus (Herz et al., 2018). It has been proposed that 130 Hz-DBS may interfere with the delaying impact of STN on this “race” between the competing inputs by disruption or at least modulation of theta-mediated pathways between midfrontal regions and STN (Cavanagh et al., 2011, Jahanshahi et al., 2015).

Yet, the probability of an error was not altered with 130 Hz-DBS, and thus, the reversed relationship between midfrontal theta and antisaccade latency was not linked to impulsive actions on a behavioral level in our study. Indeed, some studies also reported higher midfrontal theta power scaling with speeding of responses in healthy individuals under no influence of any intervention like DBS (Valadez and Simons, 2018). Here, faster response times have been interpreted as a reflection of more effective use of cognitive control. In any case, our results suggest that the mere modulation of theta-mediated midfrontal activation with high frequency DBS may not in itself be a sufficient prerequisite to elicit impulsive actions when the task remains unambiguous and without conflict throughout trials. It remains to be further investigated which task demands or individual differences in either lead location or DBS settings beyond stimulation frequency may promote or even prohibit impulsive actions.

### 4.6 Limitations

A major limitation of our study is its small sample size which may constrain generalizability of our results requiring confirmation in larger studies in the future. However, recruitment of eligible participants with PD and STN-DBS without any exclusion criteria is inherently very limited. Additionally, the study protocol was challenging to complete for this population as supported by the high proportion of pre-mature withdrawals of 44 % despite careful screening of potential participants.

Since we did not include a healthy control group, we cannot state that response inhibition was overall impaired in the PD group. However, the mean antisaccade error rate of 47.3 % in off DBS-state is within the range of comparable studies in PD and considerable higher than in healthy age-matched controls (Waldthaler et al., 2021).

Participants completed the study in off-medication state. While this is a clear advantage of the study since it excludes effects of dopaminergic medication on the results to a large extent (long lasting effects >12 h cannot be entirely excluded), dopamine replacement therapy and DBS may interact in their effects on impulsivity in PD in real life scenarios. For instance, Bakhtari and colleagues showed that dopamine replacement therapy partly restored the detrimental effect of STN-DBS on antisaccade error rates (Bakhtiari et al., 2020).

Participants were stimulated with their individual optimal DBS program and amplitude, impulse width, and DBS contacts were not changed for the study. Thus, DBS settings were not standardized between participants. On the other hand, standardization of DBS settings would have carried a high risk for side effects since therapeutic and side effects of DBS vary widely between patients and optimal settings are the result of highly individualized programming procedures. Further, additional factors such as individual deviations from optimal lead placement could have not been standardized anyway. By keeping the individualized optimal DBS settings instead (other than frequency), we aimed to avoid side effects and to resemble the DBS effect achieved in daily life with chronic stimulation.

Since all conditions were recorded on the same day, carry-over effects of DBS cannot be completely excluded. However, any risk of systematic bias caused by carry-over effects was counteracted through randomizing the order of conditions.

TEED was not kept constant between the DBS settings used in the study. TEED is expected to be lower with 60 Hz than with 130 Hz stimulation when stimulation amplitude is kept constant. As a recent study showed that changing DBS amplitude influences antisaccade performance (Munoz et al., 2021), we cannot exclude that any performance differences may be related to TEED differences (see also Methods section for further elaboration on this issue).

The small trial numbers impeded the inclusion of error trials as a control condition in the analysis of the EEG data. Thus, we cannot be certain that the increasing effect of 60 Hz-DBS on preparatory midfrontal theta power was limited to trials in which the inhibition of an impulsive response succeeded later in the trial. Given the low numbers of participants and of trials per participant, the results of this study should be considered preliminary requiring replication in larger samples.

## 5 Conclusions

In summary, a combined approach of eye-tracking and high-density EEG allowed us to differentiate the effects of two commonly used STN-DBS frequencies on response inhibition in the antisaccade task and to explore their cortical correlates. While the latency of correct antisaccades was reduced by 130 Hz and 60 Hz pulse frequencies alike, the latency of reflexive errors increased under both DBS conditions. Thus, our preliminary results do not support a general association of STN-DBS with oculomotor impulsivity. Instead, STN-DBS may promote voluntary actions at the expense of slower reflexive responses.

The probability for impulsive errors decreased exclusively with 60 Hz-DBS. As the effect size was small, it remains to be determined whether this potentially beneficial effect of mid-frequency DBS on response inhibition is reproducible and clinically relevant.

Only 130 Hz-DBS reversed the relationship between preparatory midfrontal theta activity and antisaccade latency in comparison with DBS off-state in a trial-wise analysis. Sixty Hz-DBS, on the other hand, was associated with an increase in preparatory midfrontal theta power compared with the off-DBS state which may be interpreted as a correlate for enhanced cognitive control. Hence, our results warrant future studies on the cognitive effects of mid-frequency STN-DBS in PD. Since high and mid-frequency STN-DBS may thus differentially modulate response inhibition capacity, pulse frequency needs to be considered when interpreting inconclusive results of previous and upcoming studies on the effects of STN-DBS on cognitive control.

## Funding

This study was supported by the SUCCESS program of Philipps-University Marburg (JW), the Hessian Ministry of Sciences and the Arts, clusterproject: The Adaptive Mind – TAM (FB / AK) and the German Research Foundation (DFG). International Research Training Group 1901 (FB / AK).

## Financial disclosures

D.P. and L.T. received payments as a consultants for Boston Scientific. L.T. received honoraria as a speaker on symposia sponsored by Boston Scientific and Medtronic. J.W., A.S., A.K., C.S. & F.B. have nothing to report.

## CRediT authorship contribution statement

**Josefine Waldthaler:** Conceptualization, Methodology, Software, Investigation, Data curation, Formal analysis, Visualization, Funding acquisition, Writing – original draft. **Alexander Sperlich:** Project administration, Software, Investigation, Data curation, Formal analysis, Writing – review & editing. **Aylin König:** Project administration, Software, Investigation, Data curation, Formal analysis, Writing – review & editing. **Charlotte Stüssel:** Project administration, Software, Investigation, Data curation, Formal analysis, Writing – review & editing. **Frank Bremmer:** Resources, Supervision, Funding acquisition, Writing – review & editing. **Lars Timmermann:** Resources, Supervision, Funding acquisition, Writing – review & editing. **David Pedrosa:** Conceptualization, Methodology, Software, Data curation, Project administration, Supervision, Writing – review & editing.

## Declaration of Competing Interest

The authors declare that they have no known competing financial interests or personal relationships that could have appeared to influence the work reported in this paper.

## Data Availability

The raw datasets are available upon reasonable request from the corresponding author. Newly generated code for the task and analysis pipelines is available on https://github.com/JoWld/PD_DBS_Antisac
