# Supplementary material for: High (130 Hz)- and mid (60 Hz)-frequency deep brain stimulation in the subthalamic nucleus differentially modulate response inhibition: A preliminary combined EEG and eye tracking study
Source: Neuroimage Clin. 2023 Jan 5;37:103314. doi: 10.1016/j.nicl.2023.103314 (PMC9841351; doi:10.1016/j.nicl.2023.103314)
Supplement: Supplementary Data 1 [file mmc1.docx]

Supplementary Material

**High (130 Hz)- and Mid (60 Hz)-Frequency Deep Brain Stimulation in the Subthalamic Nucleus differentially modulate Response Inhibition in Parkinson’s Disease**

**SUPPLEMENTARY METHODS**

**Sample size calculation**

We based our sample size calculation on the literature about DBS effects on antisaccades in PD identified in the recent meta-analysis. Here, eight studies reported antisaccade latency in PD on and off DBS, while only five studies reported error rates. The average Cohen’s d effect size for antisaccade latency was 0.44 (95%-confidence interval (CI) = [-0.90; -0.09]), respectively 0.50 (CI = [-0.08; 1.07]) for error rate. With the effect size of 0.44 (0.5), to detect a mean difference in a one-way repeated measures analysis of variance (RM-ANOVA) with three conditions (off / 130 Hz / 60 Hz) and a two-sided significance level of 5%, power of 90% and assumed correlation between repeated measures of 0.5 would require a total sample size of 13 (11) participants. Anticipating a drop-out rate of 25 %, we aimed for recruitment of 18 participants.

| **ID** | **left STN active contacts** | **left STN amplitude (mA)** | **left STN impulse width (µs)** | **left STN frequenzy (Hz)** | **right STN active contacts** | **right STN amplitude (mA)** | **right STN impulse width (µs)** | **right STN frequency (Hz)** |
| --- | --- | --- | --- | --- | --- | --- | --- | --- |
| 1 | 1- (34%), 2- (34%, 3- (34%),  4- (34%), 5- (34%), 6- (33%),  7- (33%) | 1.8 | 50 | 130 | 1- (33%), 2- (33%), 3- (33%), 4- (33%), 5- (34%) | 1.8 | 50 | 130 |
| 2 | 2- (60%), 15- (40%) | 2.2 | 60 | 130 | 2- (40%), 5- (10%), 7- (10%) | 1.9 | 60 | 130 |
| 3 | 1- (20%), 2- (28%), 3- (26%),  4- (26%) | 0.5 | 60 | 130 | 5- (34%), 6- (33%), 7- (33%) | 1 | 60 | 130 |
| 4 | 2- (5%), 3- (3%), 4- (12%),  5- (22%), 6- (11%), 7- (47%) | 2.0 | 60 | 130 | 1- (10%), 2- (6%), 3- (24%),  4- (60%) | 3 | 60 | 130 |
| 5 | 2- (34%), 3- (33%), 4- (33%) | 2.0 | 60 | 130 | 2- (34%), 3- (33%), 4- (33%) | 1.7 | 60 | 130 |
| 6 | 5- (33%), 6- (33%), 7- (33%) | 1.2 | 60 | 130 | 5- (33%, 6- (33%), 7- (33%) | 1.2 | 60 | 130 |
| 7 | 1- (10%), 2- (84%), 3- (3%),  4- (3%) | 0.9 | 60 | 130 | 2- (54%), 4- (29%), 3- (17%) | 0.6 | 60 | 130 |
| 8 | 2- (60%), 4- (15%), 7- (25%) | 1.5 | 60 | 130 | 3- (40%), 4- (40%), 5- (20%) | 1.5 | 60 | 130 |
| 10 | 2- (33%, 3- (33%), 4- (33%) | 1.7 | 90 | 130 | 2- (33%), 3- (33%), 4- (33%) | 1.2 | 90 | 130 |
| 11 | 3- (20%), 4- (20%), 5- (40%),  6- (20%) | 2.5 | 60 | 130 | 1- (5%), 3- (20%), 4- (20%), 5- (55%) | 1.2 | 60 | 130 |
| 12 | 2- (5%), 5- (60%), 6- (35%) | 3.8 | 60 | 130 | 3- (25%), 5- (15%), 7- (30%), 8- (30%) | 1.6 | 60 | 130 |
| 13 | 5- (4%), 6- (18%), 7- (18%),  8- (60%) | 2.2 | 60 | 130 | 5- (26%), 6- (7%), 6- (7%),  8- (60%) | 2.2 | 60 | 130 |
| 14 | 2- (10%), 3- (10%), 5- (10%),  6- (10%), 4- (30%), 7- (30%) | 1.0 | 50 | 130 | 2- ((10%), 6- (10%), 7- (10%), 5- (20%), 3- (20%), 4- (30%) | 1.7 | 50 | 130 |

**Individual DBS programs**

**Suppl-Table 1:** Individual DBS programs based on optimal clinical effects. Please see Figure Supp1 for nomenclature for the contacts of the directional DBS lead.


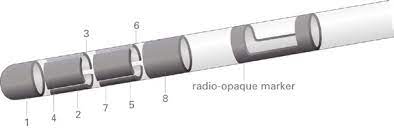


**Suppl-Figure 1:** DBS lead design with nomenclature of the contacts as refence for Suppl-Table 1 (https://www.bostonscientific.com/content/dam/Manuals/us/current-rev-en/92104398-01_Vercise™_DBS_Leads_DFU_en-USA_s.pdf [April 7, 2022]).

**SUPPLEMENTARY RESULTS**

**Preparatory EEG dynamics in the lateral prefrontal ROI**

**
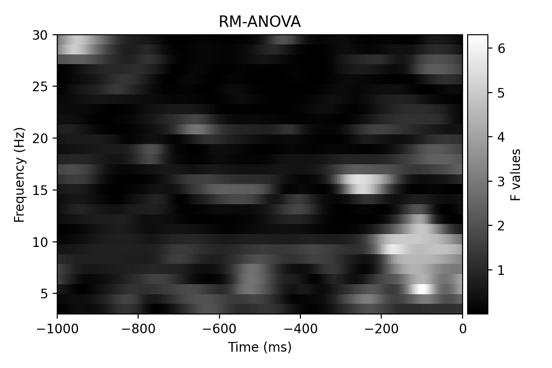

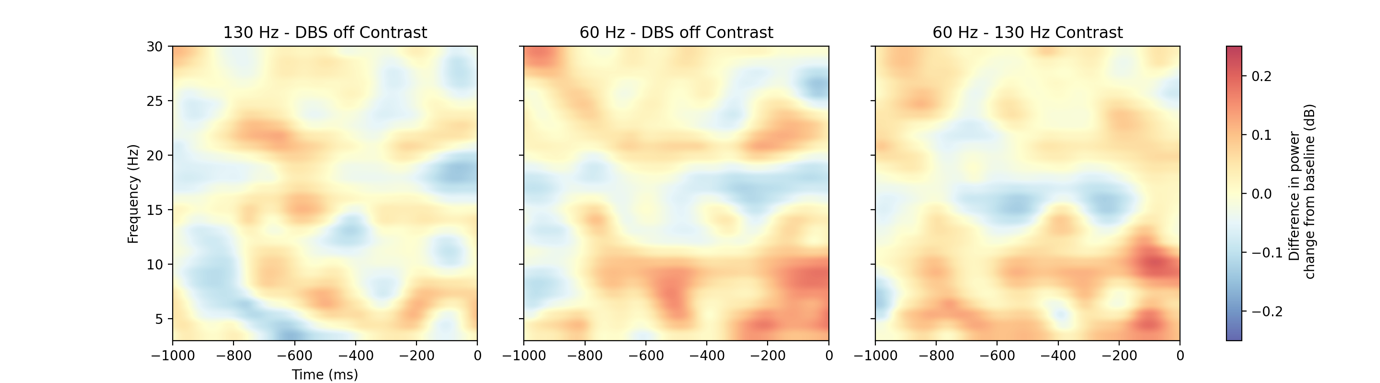
**

**Suppl-Figure 4:** Upper row: Results of the RM-ANOVA comparing the averaged time-frequency representations in the right lateral prefrontal region of interest (AF8, F6, F8, and FC6) between the three DBS condition. Non-significant F values in sequential gray, no cluster reached statistical significance. Lower row: Time-frequency representations of the contrast between conditions. No significant clusters in the pairwise comparisons.

**Additional single-trial EEG analyses**

**Antisaccade latency – midfrontal preparatory beta power**

In the linear mixed model evaluating the relationship between midfrontal beta power, DBS condition and antisaccade latency, we observed a main effect of condition on antisaccade latency as expected from behavioral findings (χ²(2) = 32.881, p < 0.001) with significant differences between 130 Hz DBS and off-DBS state (β = 0.236, 95%-CI = [0.15, 0.32], t(1654) = 5.529, p_adj_ < 0.001) and between 130 Hz DBS and 60 Hz DBS (β = 0.165, 95%-CI = [0.09, 0.24], t(1654) = 4.135, p_adj_ < 0.001). There was no main effect of beta power (χ²(1) = 1.247, p = 0. 0.3), nor a significant interaction effect between condition and beta power (χ²(2) = 4.650, p = 0.1).

**Antisaccade accuracy – midfrontal preparatory beta power**

In the general mixed model evaluating the relationship between midfrontal beta power, DBS condition and antisaccade accuracy, we observed a main effect of condition on antisaccade accuracy (χ²(2) = 10.371, p = 0.006) with significant differences between 130 Hz DBS and 60 Hz DBS state (β = -0.343, z = -3.221, p_adj_ = 0.002). There was no main effect of beta power (χ²(1) = 0.444, p = 0.5), nor a significant interaction effect between condition and beta power (χ²(2) = 1.290, p = 0.5).

**Antisaccade accuracy – midfrontal preparatory theta power**

In the general mixed model evaluating the relationship between midfrontal theta power, DBS condition and antisaccade accuracy, we observed a main effect of condition on antisaccade accuracy (χ²(2) = 10.742, p = 0.005) with significant differences between 130 Hz DBS and 60 Hz DBS state (β = -0.354, z = -3.309, p_adj_ = 0.02). There was no main effect of theta power (χ²(1) = 0.123, p = 0.7), nor a significant interaction effect between condition and theta power (χ²(2) = 2.637, p = 0.3).
